# The Medical Commencement

**Published:** 1883-03

**Authors:** 


### The Medical Commencement.

The commencement exercises of the Medical College were held at St. James Hall on Tuesday evening, Feb. 27, 1883.

Prof. M. D. Mann delivered the address to the graduates, and Hon. James M. Smith, Judge of the Superior Court of Buffalo, spoke to the alumni. The address of Prof. Mann was eminently practical and suited to the occasion. It contained many humorous illustrations with such advice as the class of young men, just entering upon the work of the profession, require.

Judge Smith's address was such an one, as the earnest Judge, whose life is a noble example of industry and energy, would be sure to give on an occasion of this kind.

The annual banquet and reunion was held at The Genesee. The seat of honor at the head of the table was occupied by Dr. Wm. Ring, one of the oldest alumni of the college.

The following toasts were proposed, each eliciting a happy response:

"Our Beloved Alma Mater." Response by Dr. Thomas F. Rochester.

"The Medical Profession," Response by Dr. Andrews.

"The Legal Profession." Response by the Hon. E. C. Sprague.

"The Clergy." Response by the Rev. G W. Cutter.

"The Alumni Association of the University of Buffalo." Response by Dr. E. N. Brush, of Utica.

"General Science." Response by the Very Rev. Dean Hart.

"The Medical Societies" Response by Dr. J. W. Keene.

"The Board of Curators of the University of Buffalo." Response by Dr. C. H. Richmond.

"Our City." Response by the Hon John B. Manning, Mayor of Buffalo.

"The Press." Response by Joseph O'Connor, Editor *Daily Courier*.

"The Ladies" Response by Dr. Lucien Howe.

"The Graduating Class of '83." Response by Dr. Wm. A. Gray.

